# Current concepts in the diagnosis and management of Os Acetabuli

**DOI:** 10.1007/s00264-023-06078-0

**Published:** 2024-01-10

**Authors:** Junya Yoshitani, Benjamin Schoyer, Anand Shah, Vikas Khanduja

**Affiliations:** 1 Young Adult Hip Service, Addenbrooke’s - Cambridge University Hospitals NHS Foundation Trust, Cambridge, CB2 0QQ UK; 2 School of Clinical Medicine, University of Cambridge, Cambridge, UK

**Keywords:** Os acetabuli, Hip arthroscopy, Dysplasia, Femoroacetabular impingement

## Abstract

**Purpose:**

The aim of this review is to appraise the current evidence on the epidemiology, pathophysiology, diagnosis and management of os acetabuli.

**Methods:**

A scoping review was conducted according to the Joanna Briggs Institute guidelines. A systematic search was performed on Medline (PubMed), Embase and Cochrane Library. Inclusion criteria comprised observational and interventional studies and review articles published in the English language that focused on patients with os acetabuli according to the PRISMA extension of scoping reviews checklist using the terms ‘Os Acetabuli’ or ‘os acetabula’ or ‘acetabular ossicles’. A narrative synthesis of results was undertaken, and the included articles were divided into (i) definition, (ii) aetiology, (iii) diagnosis and imaging and (iv) management of os acetabuli.

**Results:**

107 articles were screened, with 22 meeting the eligibility criteria. A total of 8836 patients were considered, of which 604 had os acetabuli. The mean age was 32.8 years. The prevalence of os acetabuli ranged from 3.4 to 7.7%, with a higher prevalence in males compared to females. True os acetabuli was defined as an unfused secondary ossification centre along the acetabular rim. The aetiology of os acetabuli is thought to be secondary to acetabular dysplasia and/or femoroacetabular impingement. Standard of care for management of symptomatic os acetabuli is considered to be arthroscopic excision unless the excision results in acetabular undercoverage and/or instability, in which case, fixation is recommended.

**Conclusions:**

Successful management of os acetabuli depends on understanding the pathology and treating the underlying cause rather than treating the os acetabuli in isolation. Future work needs to focus on establishing clear diagnostic criteria, consensus on definition and an evidence-based treatment algorithm.

## Introduction

**Fig. 1:**
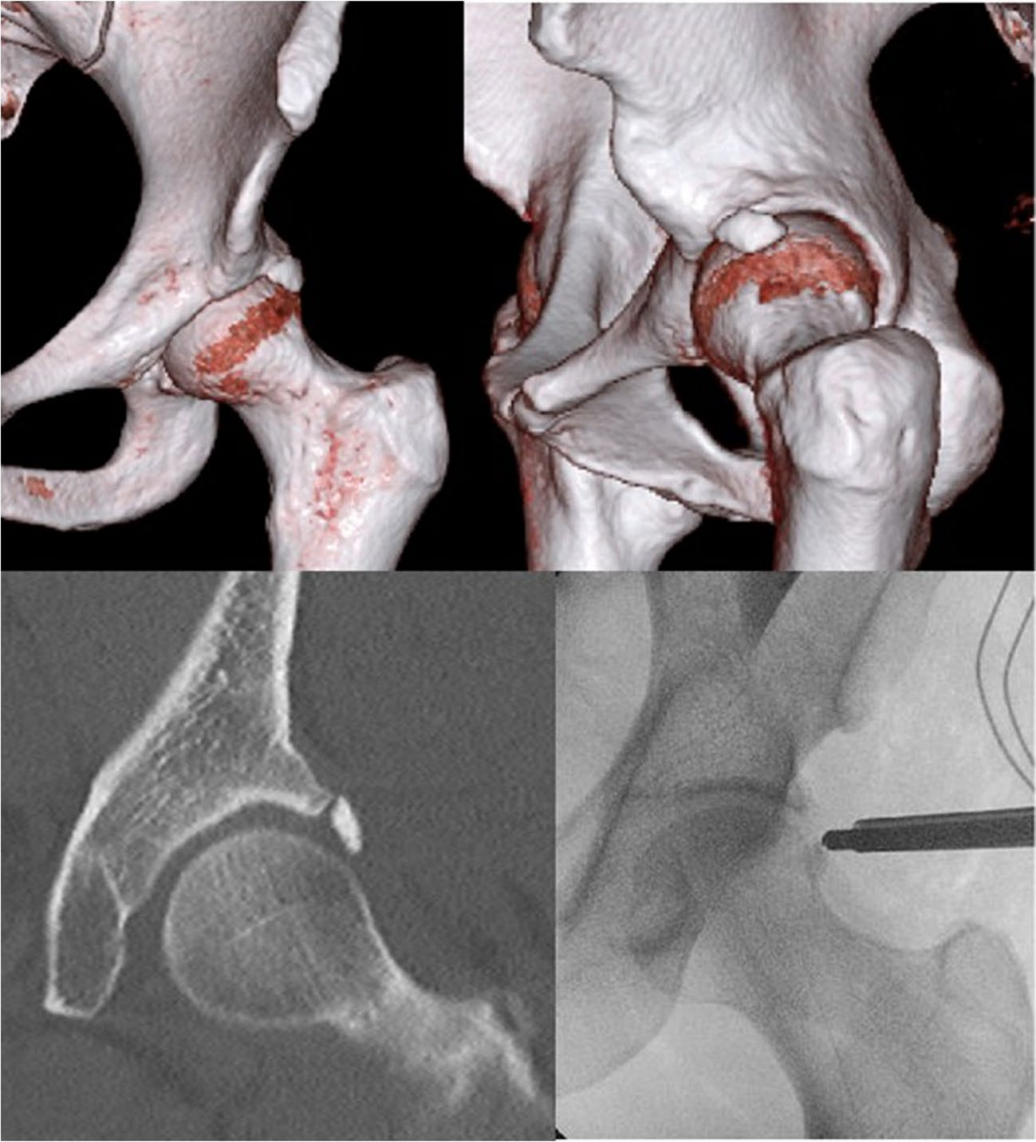
A case of os acetabuli: The upper row depicts a 3D-CT reconstruction of os acetabuli. The bottom left illustrates os acetabuli in a coronal plane of CT. The bottom right displays the arthroscopic resection of os acetabuli

Hip arthroscopy is an effective surgical intervention for patients with symptomatic femoroacetabular impingement (FAI) and other acetabular rim pathologies [1–3]. Due to the rising number of hip arthroscopy procedures being performed, os acetabuli is increasingly recognised as a potential cause of hip pain and reduced function [4, 5]. Os acetabuli are small ossicles near the acetabular rim that may occur due to an unfused secondary ossification centre, rim fractures, FAI or labral calcifications (Fig. 1) [6, 7]. Although several studies have been published on os acetabuli in recent years, there is a lack of consensus on the definition and approach to management. An improved understanding of the aetiology, pathology and an evidence-based approach to management is required to provide optimal care. The aim of our scoping review, therefore, was to appraise the current evidence regarding os acetabuli, with a focus on the pathophysiology and existing methods to diagnose and manage the condition.

### Objectives

To guide the writing of this scoping review, the authors used the population, concept and context approach (PCC). Original articles on os acetabuli were evaluated.

PCC framework was used to formulate the questions:

P (*population*): patients with os acetabuli.

C (*concept*): to better understand the consequences of pathophysiology and subsequent management.

C (*context*): No particular setting or location.

The clinical questions are (a) What is the definition of os acetabuli, (b) What is the aetiology of os acetabuli, (c) How is os acetabuli diagnosed and (d) How should patients with os acetabuli be managed?

## Methods

### Protocol

The scoping review was performed according to the Joanna Briggs Institute guidelines [8].

The Preferred Reporting Items for Systematic Reviews and Meta-Analyses extension for Scoping Reviews (PRISMA-ScR) checklist was used to report the results [9].

### Eligibility criteria

Inclusion criteria comprised observational and interventional studies and review articles published in the English language that focused on patients with os acetabuli and its pathophysiology. Exclusion criteria included protocol studies, abstract-only publications, letters, conference articles and textbooks.

### Search strategy and information sources

A comprehensive search of the literature on Medline (PubMed), Embase and Cochrane databases was performed on 11th June 2023. The following keywords were used: (‘Os Acetabuli’[All Fields]) OR (‘os acetabula’[All Fields]) OR (‘acetabular ossicles’[All Fields]. Reference list checking was also performed using articles identified by the above search process.

### Selection of sources of evidence

Search results from the multiple databases were imported into the Rayyan systematic reviews web application (Qatar Computing Research Institute, Doha, Qatar) for screening and selection of relevant articles [10]. Two authors (JY and BS) independently screened the titles and abstracts for eligibility, followed by the full texts in a three-stage determination process. Disagreements between the two reviewers were resolved by a third senior author (VK).

### Data charting process

Two reviewers (JY and BS) independently extracted data from each eligible article onto pre-determined forms. The data extracted included the following.

### Data items

The data extraction form contained the first author, year of publication, type of study, number of patients, characteristics of patients (gender and mean age) and the information of os acetabuli.

### Synthesis of results

A narrative synthesis of results was undertaken, and the included articles were divided according to the following categories: (i) definition, (ii) aetiology, (iii) diagnosis and imaging and (iv) management.

## Results

### Selection of sources of evidence

**Fig. 2:**
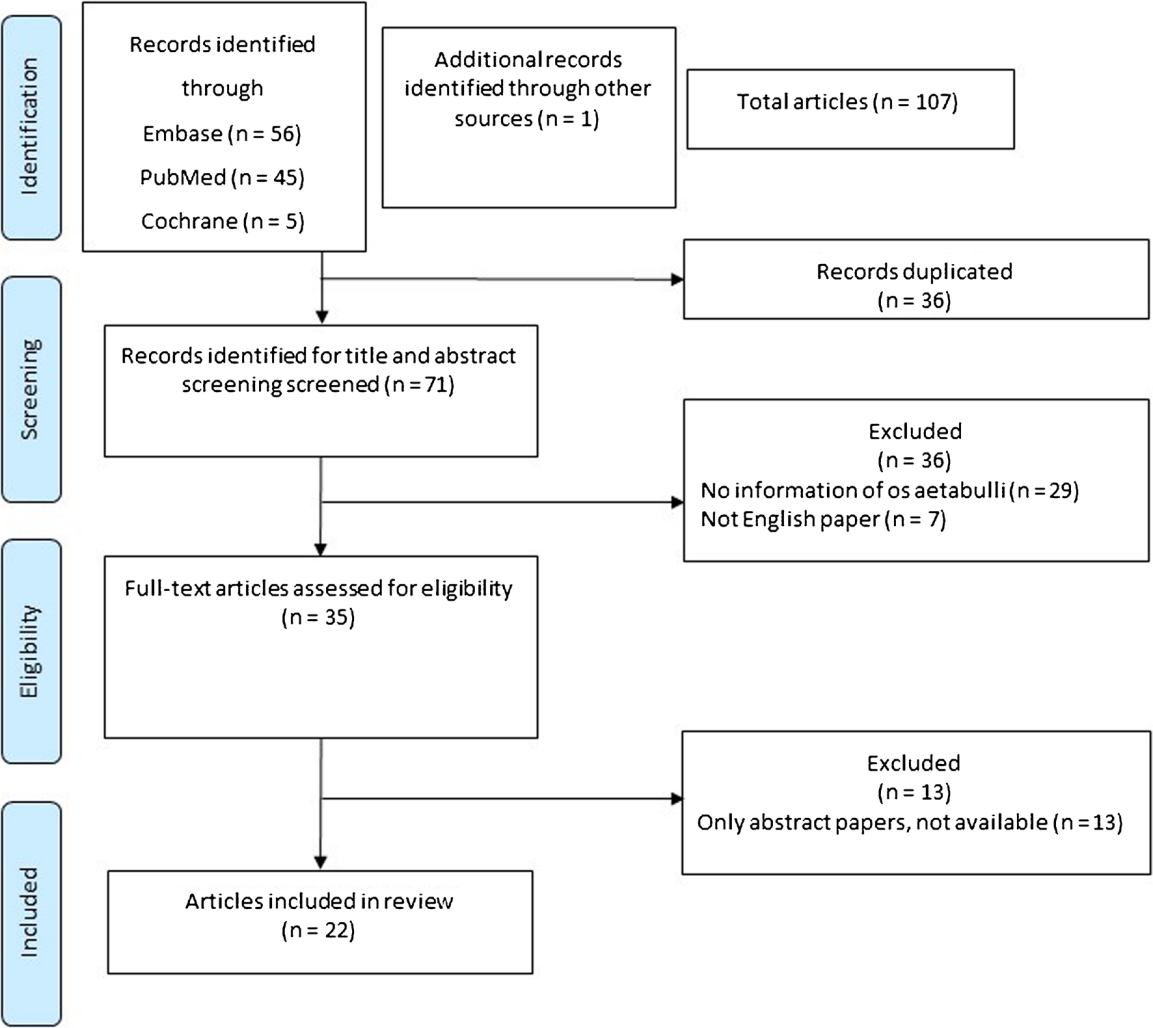
Flow diagram of included and excluded publications

The literature search resulted in 107 articles, from which 36 articles were excluded because of duplication. Furthermore, 36 articles were excluded following title and abstract screening, leaving 35 articles for full-text analysis. Thirteen articles were excluded because they were only available as abstracts without full-text access, leaving 22 citations that met the inclusion criteria for the scoping review. A summary of this process is provided in Fig. 2.

### Characteristics of evidence sources

Of the included articles, ten were case reports or articles outlining surgical technique (10/22), seven case series (7/22), three retrospective case–control studies (3/22), one retrospective cohort study (1/22) and one review. Three articles reported on terminology, six articles referred to aetiology, five articles pertained to diagnosis and 14 to management, although overlap was present. Table 1 presents an overview of the included papers, excluding those referring to surgical technique and the review. A total of 8836 patients were included in this study, of which 604 had os acetabuli. The average age was 32.8 years.

**Table 1:** Overview of included studies

First author	Year	Os acetabuli patients	Female	Male	Pathology	The number of all patients	Mean age
Klaue, K	1991	9	N/A	N/A	Dysplasia	24	N/A
Plotz, G. M. J	2002	1	1	0	Dysplasia	1	14
Kassarjian, A	2005	17	N/A	N/A	Cam-type FAI	40	36.5
Martinez, A. E	2006	15	3	12	FAI	495	30
Kumar, J	2007	1	0	1	Labral ossification	1	45
Singh, P. J	2010	2	0	2	Football player who had hip arthroscopy	24	22
Larson, C. M	2011	2	0	2	FAI	2	22
Aprato, A	2013	8	N/A	N/A	FAI	41	24
Jackson, T. J	2014	94	N/A	N/A	Os acetabuli and amorphous calcific deposit	1872	N/A
Cuellar, A	2015	1	1	0	Dysplasia	1	42
Brian D Giordano	2017	21	6	15	Rim fracture	21	33
Yoshi Pratama Djaja	2019	403	N/A	N/A	FAI	5948	43.9
Randelli Filippo	2019	21	1	20	FAI	273	31
Lund, B	2021	3	0	3	Soccer and handball players	3	21
Salvador, J	2022	6	0	6	Soccer players	90	25.8

*FAI*, femoroacetabular impingement

### Terminology and definition

**Fig. 3:**
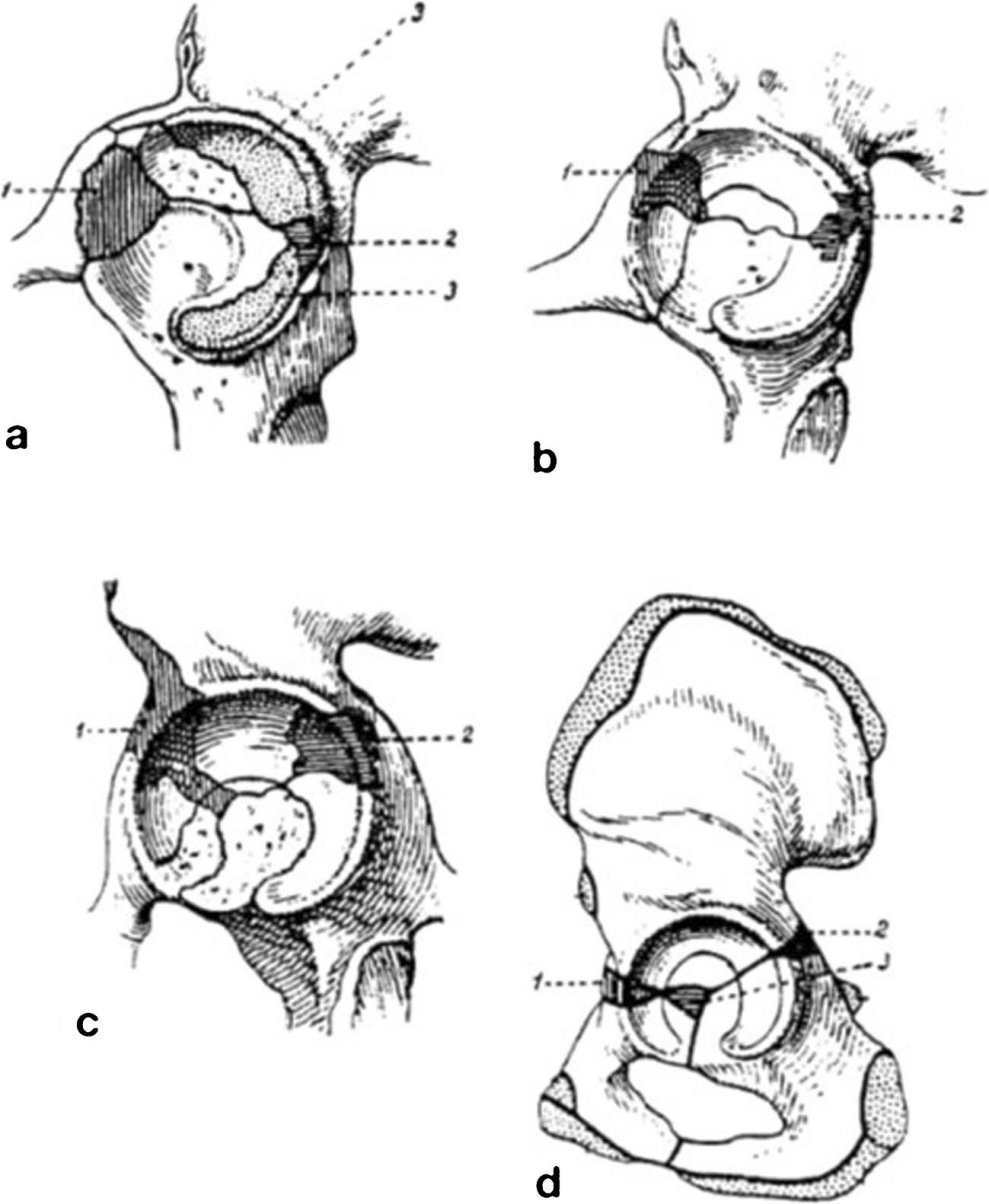
(a–d) Sketch of accessory ossification centres of the hip (referred by Hergan et al.): (1) os acetabuli anterius, (2) os acetabuli posterius and (3) further plate-like ossification centres (including os acetabuli centrale in d)

Klaue et al. referred to articles by Albinus from 1737, which described the remnants of secondary centres of ossification, and Klaue proposed the term ‘os acetabuli’ in 1876 [11]. The occurrence of fragments during later stages and their prolonged existence have been noted in cases of unfused secondary ossification nucleus, rim fractures, labrum calcifications, rickets, osteomyelitis, tuberculosis and osteochondritis dissecans [11, 12]. Fragments have also been attributed to excessive stress on the acetabular rim in a dysplastic joint, resulting in the fracturing and subsequent separation of a segment of the rim [11]. The most commonly described ossicle is os acetabuli anterius, also known as os supertilii, os ad acetabulum, os marginale superius acetabuli and os coxae quartum [6, 13]. It is situated in the anterosuperior margin of the Y-cartilage, between the os ilium and pubis. Additionally, os acetabuli posterius has been described, also referred to as noduli, which is located in the posterosuperior or posterior apophyseal cartilage (Fig. 3) [6, 13]. In addition to the clear aetiology and pathophysiology of os acetabuli, there are numerous uncertainties and debates regarding the diagnostic features of these lesions [12]. As a result, true os acetabuli has been defined in previous studies as an unfused secondary ossification centre of the acetabulum among the various radiopaque structures observed around the acetabular rim [12, 14, 15].

### Aetiology

**Fig. 4:**
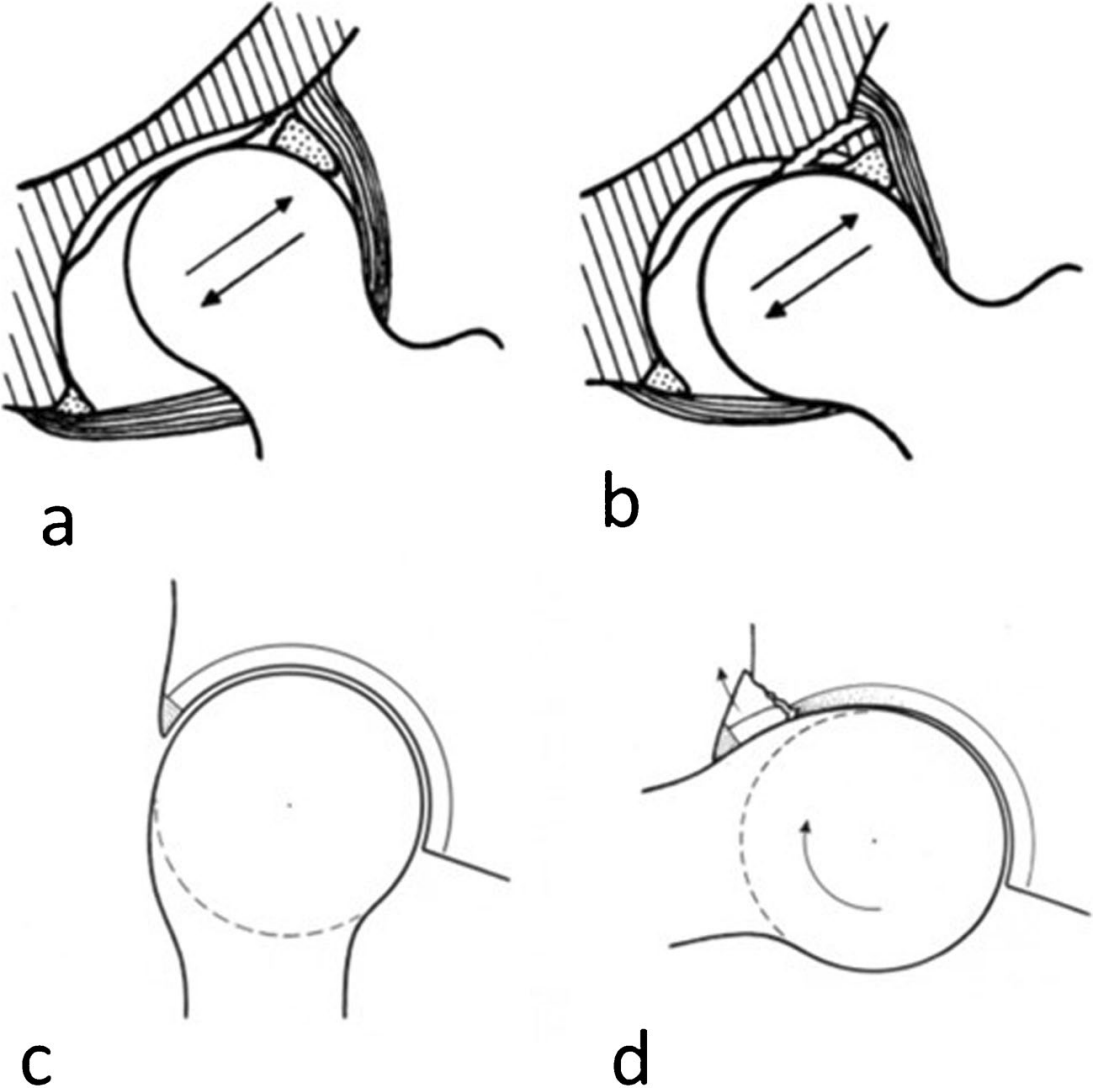
Schematic figures showing the mechanism that leads to a fatigue fracture of the acetabular rim. (**a**) and (**b**) were referred to by Klaue et al. [11]. (**a**) In type 1 dysplasia hip, the labrum is overloaded and may shear from the acetabular bony rim. (**b**) In type 2 dysplasia hip, a fatigue fracture may occur, causing os acetabuli. (**c**) and (**d**) were referred to by Martinez et al. [14]. The aspheric part of the head-neck junction squeezes into the retroverted part of the acetabulum and eventually leads to a fatigue fracture of the rim. The adjacent cartilage is also compressed

It has been observed that the bone fragment of the acetabular rim can be seen around 6 months after birth and typically disappears before the age of 20 [11, 15]. Klaus demonstrated that all cases of acetabular rim lesions exhibited reduced acetabular coverage of the femoral head [11]. Dysplastic hips were categorised into two types: type 1 hips are characterized by a shallower and more vertically oriented acetabulum than normal, and type 2 hips have an acetabulum with inadequate coverage of the femoral head (‘short roof’) and a curvature radius similar to that of the femoral head (Fig. 4). In type 2 hips, the relatively shorter acetabular roof decreases the loaded surface area, resulting in elevated pressure, especially at the margin of the acetabulum. This increased pressure may lead to stress fractures and the separation of a fragment from the rim [11]. However, Plotz et al. proposed an additional explanation, hypothesising that the chronic shearing forces resulting from acetabular dysplasia contributed to the persistence of this bony fragment [16]. Hergan et al*.* listed diseases resembling acetabular ossicles: osteochondrosis dissecans, posttraumatic intra-articular bone fragments and degenerative disease [6]. The authors suggested that confusion may occur when a traumatic fracture of an osteophyte occurs, closely resembling a persistent os acetabuli or a loose articular body [6]. According to Martinez et al. [14], the formation of this acetabular rim fragment is believed to be caused by fatigue resulting from FAI. The non-spherical part of the hip joint’s femoral head is wedged into the acetabulum and gradually leads to stress fractures in the retroverted portion of the acetabulum (Fig. 4). Although true ‘os acetabuli’ share similar morphology, the orientation of the cartilaginous growth plate is more parallel to the joint surface in contrast to rim fractures, where the separation line is perpendicular to the joint surface [14].

A recent case–control study demonstrated a prevalence of 8.65% (25/289) for ossicles in the symptomatic group and 3.33% (378/11,356) in the asymptomatic group [12]. The types of ossicles found in the general population were predominantly labral calcifications (55.09%), followed by rim fractures (35.73%), unfused ossification centres (1.24%) and loose bodies (7.94%) [12]. Labral calcifications, which were mostly asymptomatic, exhibited the smallest-sized ossicles. The size of ossicles was found to be associated with symptoms (895.28 vs. 103.64 mm^3^; *p* < 0.001) [12]. The researchers concluded that the overall prevalence of os acetabuli was 3.46%, and they identified a significant association between rim fractures and FAI (83.33%; *p* < 0.001), but not with any specific types of FAI [12]. Several studies have documented the occurrence of os acetabuli in patients with symptomatic FAI, with reported incidences ranging from 3.6 to 7.7% [14, 17–19]. These studies also investigated the association between os acetabuli and the presence of symptomatic FAI syndrome. Identifying the type of acetabular ossicles can be challenging, particularly when distinguishing between an unfused secondary ossification centre (true os acetabuli) and a rim fracture [12]. In such cases, the location and appearance of the lesion can assist in determining its type. If the lesion is found in the superior half of the acetabulum and not in the anterior quadrant, it is most likely to be a rim fracture rather than a true os acetabuli since this location is uncommon for a true os acetabuli [12]. The occurrence of acetabular ossicles in the male population was found to be 2.2 times higher than in the female population [12]. This finding is comparable with previous studies investigating acetabular ossicles. For example, Martinez et al*.* identified only three female patients among a total of 15 cases [14], while Randelli et al*.* reported just one female patient out of 21 cases of os acetabuli [17].

### Imaging

Symptomatic patients with an acetabular ossicle should be worked up with a plain radiograph followed by a computed tomography (CT) scan [6]. Djaja et al. reported that the use of CT increases the sensitivity of detection of the acetabular ossicle [20]. Martinez et al. reported that preoperative magnetic resonance imaging (MRI) in patients with os acetabuli revealed the presence of the fragment consisting of the labrum, articular cartilage and bone [14]. Low signal intensity in proton density-weighted images revealed that the inner aspect of the fragment had sclerotic margins. The gap between the fragment and surrounding tissue was filled with connective tissue. Additionally, using magnetic resonance arthrography (MRA), Randelli et al*.* reported that it was possible to observe a close association between os acetabuli and both the articular cartilage and labrum [17]. Alessandro et al*.* conducted a study evaluating the diagnostic correlation between MRA and intraoperative findings [21]. MRA exhibited 100% sensitivity, specificity, positive predictive value (PPV) and negative predictive value (NPV) of 100% for detecting an os acetabuli [21]. Jackson and colleagues compared the features of labral calcifications and os acetabuli acetabuli [22]. Labral calcifications were distinct from os acetabuli in that they did not have any trabecular or cortical bone and were much smaller in size. Additionally, they had poorly defined borders and exhibited more opacity compared to adjacent trabecular bone [22].

### Management

Treatment options for os acetabuli, FAI and dysplasia have evolved over recent years. In 2006, Martinez et al*.* performed a study involving 18 hips with os acetabuli and FAI that underwent surgical dislocation of the hip for excision of acetabular rim fragments and osteochondroplasty of the femoral head-neck [14]. Since 2010, most studies evaluating the treatment of os acetabuli have reported on outcomes following arthroscopic hip surgery.

A retrospective case series reported on the intra-articular hip pathologies and treatment of 24 consecutive Australian football league players (27 hips) who had arthroscopic hip surgery for groin pain [23]. The least frequent abnormality was the presence of loose os acetabuli, which was found in only 7% of the players and was surgically removed [23]. All players resumed full training for three months. In another case series, soccer players who underwent arthroscopic excision of os acetabuli were able to return to their previous level of competition without requiring any further treatment at a one-year follow-up [19].

Larson et al. [24] reported on two cases where large rim fractures and/or os acetabuli contributed to both FAI and instability. As complete excision would result in potential dysplasia and instability, they performed partial resection and internal fixation with arthroscopic-assisted cannulated screws. Healing of the stabilised rim fragment and improved patient outcome scores was reported at a two year follow-up. The procedure was technically feasible to perform arthroscopically, and the fragment healed without formal debridement of the fibrocartilaginous component of the rim fracture [24].

A treatment algorithm for os acetabuli has been proposed based on centre-edge (CE) angles [5]. Excision of os acetabuli in patients with mild hip dysplsia results in instability and rapid development of hip osteoarthritis [25]. Complete removal is recommended in cases where the CE angles are satisfactory, regardless of the presence of the fragment (lateral CE angle > 20–25°, anterior CE angle > 20°). Where the CE angle measures less than 20–25° on coronal imaging (anteroposterior pelvis) and less than 20° on a false-profile view, partial excision and internal fixation of the remaining portion are to be considered. If the fragment is crucial for maintaining normal coverage of the femoral head and the hip would become dysplastic following excision, fixation of the entire fragment is recommended [5]. However, the authors concluded that the presence or absence of an acetabular rim fracture does not appear to affect patient-reported clinical outcomes after a minimum of two year follow-up post-arthroscopic hip surgery [5].

Randelli et al. [17] reported excellent outcomes after arthroscopic treatment of os acetabuli and FAI during a follow-up period of two years and seven months. Interestingly, arthroscopic os acetabuli excision or fixation with FAI treatment was reported to have better outcomes than FAI treatment alone [17]. The treatment outcomes were summarised in Table 2.

**Table 2:** The clinical outcome of arthroscopic surgery for os acetabuli

First author	Year	Treatment method	Outcome	Preoperative	Final follow-up	Outcome	Preoperative	Final follow-up
Singh, P. J	2010	Excision	MHHS	86	96	NAHS	81	96
Larson, C. M	2011	Partial resection and internal fixation	MHHS	81.2	98.9	SF-12	92.1	97.1
Brian D Giordano	2017	Removal or partial resection	MHHS	68.4	79.8	NAHS	65.1	79.6
Randelli Filippo	2019	Removed or trimmed, fixed with a lag screw	MHHS	57.5	95	N/A	N/A	N/A
Lund, B	2021	Internal fixation using a suture-bridge technique	HAGOS ADL	66.7	88.3	Sport	37.3	80.3
Salvador, J	2022	Arthroscopic resection	MHHS	69.7	95.7	N/A	N/A	N/A

*MHHS*, Modified Harris Hip Score; *NAHS*, Nonarthritic Hip Score; *SF-12*, Short Form 12; *HAGOS*, Copenhagen Hip and Groin Outcomes Score

Several studies and case reports have reported on techniques and outcomes following internal fixation of os acetabuli. Lund reported favourable short-term outcomes using the suture-bridge technique in young athletes [26]. In another case by Rafols et al*.* [27], a patient with bilateral rim fractures associated with FAI underwent pincer resection under X-ray guidance using power instruments, which included around 30% of the fractured segment. The remaining bone fragment was fixed using an arthroscopic-assisted 3.0-mm cannulated screw [27]. The labrum was re-attached and secured with translabral suture anchors, which covered the head of the cannulated screw.

According to Pascual-Garrido et al. [28], if the removal of these fragments leads to CEA measuring less than 25° on coronal imaging (AP pelvis) and less than 20° on a false-profile view, it is recommended to perform partial resection and internal fixation of the remaining portion [28]. In this case, the os fragment is anchored using a 3.5-mm partially threaded cannulated screw. This procedure is performed through a DALA portal [28].

Cuellar et al*.* [29] outlined an arthroscopic technique for securing os acetabuli with a compression screw. Fibrous tissue between the acetabular rim and the os acetabuli is debrided, followed by the placement of a guidewire through the os acetabuli fragment up to the acetabular rim. A cannulated screw is inserted along the wire, facilitating compression of the os acetabuli through direct bone-to-bone contact [29]. A suture-on-screw technique that mimics the function of a screw and an anchor has been described for simultaneously fixing a rim fracture and repairing a labral lesion in a patient with mixed type FAI and os acetabuli [30]. After preparation of the acetabular rim and partial excision of the os acetabuli, Carro et al*.* described the modified technique using a 4.0-mm cannulated screw for fixation of the rim fracture with a No. 2 Ultrabraid suture attached to the proximal aspect of the screw for the labral repair [30].

DeFroda et al*.* [31] described a similar suture-on-screw technique using a partially threaded cannulated screw with a washer and a nonabsorbable suture threaded through the washer, which allows the screw to function as a labral anchor specifically positioned at the 12 o’clock position [31]. Yin et al*.* introduced an all-arthroscopic method for repairing os acetabuli using absorbable anchors that penetrate the bone fragment and sutures that are tied in a double-pulley fashion [32]. The technique involved inserting two absorbable anchors to penetrate the bone fragments and secure them to the acetabular bone bed. One limb of a suture from each anchor was tied at the end and passed over the rim of the os acetabuli. The free limbs of the suture were tied down using a standard sliding knot to compress the bone fragments, employing a double-pulley technique. Another suture from the anchors was utilised to repair the labrum [32].

## Discussion

Differentiating between true os acetabuli, rim fractures, and calcifications can be challenging, but is clinically important because successful management of os acetabuli depends on understanding the pathology and treating the underlying cause. True os acetabuli has been defined as an unfused secondary ossification centre located around the rim of the acetabulum. The location and orientation of the cartilaginous growth plate can help discriminate between true and false os acetabuli. The prevalence of os acetabuli ranges from 3.46 to 7.7% and is more frequent in males. Based on the findings of this review, it is understood that os acetabuli encompasses not only true os acetabuli but also various pathologies such as rim fractures and calcifications. It is considered as a comprehensive term for bony fragments around the acetabular rim.

True os acetabuli can be secondary to both dysplasia and FAI, with the latter having received much more attention in the literature, leading to a paucity of evidence regarding the former. Klaue et al. reported nine cases of patients with dysplasia and os acetabuli and demonstrated the mechanism of stress fracture at the acetabular rim [11] (Fig. 2). In the reports by Klaue et al*.* and Plotz et al., pelvic osteotomy has been performed on patients with os acetabuli associated with dysplasia. However, Cuellar et al.excision of os acetabuli resulting in rapid progression of osteoarthritis has also been described [33]. From these observations, it is believed that treatment for os acetabuli associated with dysplasia should focus on addressing the underlying dysplasia.

A causative mechanism for rim fractures has been proposed, whereby the aspherical femoral head exerts torque while attempting to move in the acetabulum as it encounters a relatively rigid acetabular rim that has limited capacity to expand. Initially, these damage both the articular cartilage and labrum [11, 14], but the forces subsequently affect the underlying subchondral bone. Due to the application of these forces from repetitive trauma, the injury cannot repair itself quickly enough, resulting in the development of a stress fracture [14] (Fig. 2). In patients with FAI, excision or partial resection of os acetabuli has demonstrated favourable outcomes. In recent years, there has been an increasing number of reports on treatment using fixation to maintain stability. However, there is still ongoing debate regarding the comparative merits of these approaches.

Treatment is broadly categorised into excision, partial resection and fixation. In cases where the CE angle would measure less than 20–25°, fixation or partial resection is recommended for stability and adequate acetabular coverage. In recent years, surgical techniques have been developed to enable the simultaneous repair of os acetabuli and labral tears. However, all reports consist of case series or case reports without an appropriate control group or standardized outcomes, making it difficult to compare treatment outcomes. Therefore, further research using appropriate study designs is necessary in the future.

### Limitations

This scoping review has included a range of study designs and methodologies with varying levels of evidence, thus making comparisons and generalisation more difficult. Furthermore, we only included English language publications and therefore may have excluded relevant studies published in other languages. It is likely that negative and unpublished findings have been excluded due to potential publication bias. Given that the terminology surrounding os acetabuli is not yet consistent, there may have been limitations to our search strategy, especially if authors used wholly different terminology when referring to os acetabuli.

## Conclusion

An accurate diagnosis and appropriate management of os acetabuli require an appreciation of the underlying drivers of os acetabuli, namely dysplasia and FAI. The reported prevalence of os acetabuli ranges from 3.46 to 7.7%, with a higher occurrence observed in males. Standard of care for management of symptomatic os acetabuli is considered to be arthroscopic excision unless the excision results in acetabular under coverage and/or instability, in which case fixation is recommended. Future research should focus on undertaking more robust and reproducible studies to establish clear diagnostic criteria, standardised terminology and evidence-based management approaches for os acetabuli.
